# Accelerated Alzheimer’s Aβ-42 secondary nucleation chronologically visualized on fibril surfaces

**DOI:** 10.1126/sciadv.adp5059

**Published:** 2024-10-25

**Authors:** Peter Niraj Nirmalraj, Shayon Bhattacharya, Damien Thompson

**Affiliations:** ^1^ Transport at Nanoscale Interfaces Laboratory, Swiss Federal Laboratories for Materials Science and Technology, Dübendorf, CH-8600, Switzerland.; ^2^ Department of Physics, Bernal Institute, University of Limerick, Limerick V94T9PX, Ireland.

## Abstract

Protein fibril surfaces tend to generate toxic oligomers catalytically. To date, efforts to study the accelerated aggregation steps involved with Alzheimer’s disease–linked amyloid-β (Aβ)–42 proteins on fibril surfaces have mainly relied on fluorophore-based analytics. Here, we visualize rare secondary nucleation events on the surface of Aβ-42 fibrils from embryonic to endpoint stages using liquid-based atomic force microscopy. Nanoscale imaging supported by atomic-scale molecular simulations tracked the adsorption and proliferation of oligomeric assemblies at nonperiodically spaced catalytic sites on the fibril surface. Upon confirming that fibril edges are preferential binding sites for oligomers during embryonic stages, the secondary fibrillar size changes were quantified during the growth stages. Notably, a small population of fibrils that displayed higher surface catalytic activity was identified as superspreaders. Profiling secondary fibrils during endpoint stages revealed a nearly threefold increase in their surface corrugation, a parameter we exploit to classify fibril subpopulations.

## Teaser

Nanoscopic characterization of amyloid-β 42 proteins generated via secondary nucleation in a label-free manner.

## INTRODUCTION

The build-up of proteins in between brain cells by evading clearance pathways is a common denominator in several neurocognitive disorders including Alzheimer’s disease (AD) (*1*). There is a growing consensus that aggregation-prone amyloid-β (Aβ) and tau proteins are associated with AD pathologies (*2*), which need to be therapeutically targeted before symptoms become visible in individuals such as memory loss, impulsive behavior, loss of spontaneity, increased anxiety, and confusion. The clinical biomarkers associated with Aβ proteins are labeled according to the length of their amino acid sequence (i.e., Aβ-40 and Aβ-42), with the more hydrophobic and fibrillogenic Aβ-42 aggregates found in brain tissue of patients with AD (*3*). In particular, the levels of Aβ (determined by Aβ-42/40 ratio) and tau proteins (phosphorylated tau at threonine residues 217 and 181 and total tau) quantified in blood (*4*–*6*) and cerebrospinal fluid (CSF) (*7*–*9*) using biochemical assays (*10*) serve as indicators of amyloid and tau pathology. Recently, our laboratory and others have shown that the nanoscopic differences in the morphology of protein aggregates in blood and CSF also reflect the level of neurocognitive disorder in individuals spanning from subjective cognitive decline, mild cognitive impairment, and moderate to severe AD symptoms (*7*, *11*–*14*).

**Fig. 1.**
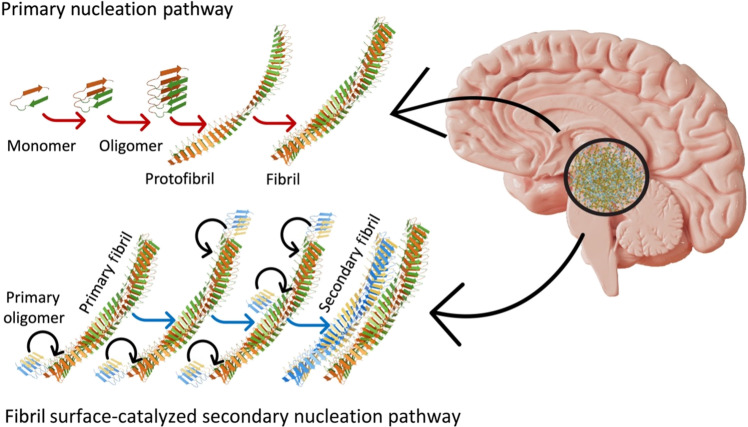
Schematic showing differences between primary and secondary amyloid nucleation pathways. The protein aggregates formed through the secondary nucleation are autocatalytically generated on the primary fibril surface. Objects are shown not to scale.

Interpreting the role of protein aggregation in AD disease progression by characterization of synthetically prepared Aβ and tau protein isoforms (aggregation initiated in physiological buffer solution) has been the subject of several in vitro studies (*15*–*28*). Primary nucleation is a process wherein monomeric forms of the proteins are mostly allowed to assemble in buffered salt solutions with well-controlled pH levels on synthetic surfaces to form oligomers and fibrils without preformed protein aggregates. Conversely, the secondary nucleation process occurs when a preformed protein aggregate such as a fibril [termed in the literature as seed, parent, or primary fibril (*15*–*18*)] formed via the primary nucleation process acts as a catalytic surface promoting the formation of new protein aggregates (*19*). Figure 1 is a schematic showing the differences between primary and secondary amyloid nucleation pathways. The energy barriers to forming new toxic oligomeric aggregates are also lower in the case of the secondary nucleation when compared to that of the primary nucleation process (*20*). How these toxic secondary oligomers accumulate and spread within human brain tissue and whether they relate to the pathology of sporadic AD remains unclear. Hence, it is becoming increasingly important to discern protein aggregates formed via the primary and secondary aggregation pathways and establish a detailed understanding of the structure-specific neurotoxicity of oligomers.

To this end, methodological advances have been able to resolve the ultrastructure of Aβ and tau protein aggregates formed along the primary nucleation pathway using cryo–electron microscopy (cryo-EM) (*21*) and solid-state nuclear magnetic resonance spectroscopy (*22*). Likewise, the aggregation kinetics and toxicity of these proteins have been investigated using ultrasensitive fluorescence-based assays (*23*), nanopore devices (*24*), and circular dichroism (*25*). Details on the morphology, on-surface assembly, and chemical structure of Aβ and tau protein aggregates have also been made accessible using standard atomic force microscopy (AFM) (*26*, *27*), video-rate AFM (*28*), and infrared spectroscopy (*29*). Although the physicochemical nature of protein aggregates formed along the primary nucleation pathway is well-studied, the morphology of transient oligomers and the mechanisms by which they are formed on fibril surfaces (secondary nucleation processes) related to Aβ and tau proteins remain to be fully clarified. This disparity stems from the limitations that existed until recently in available techniques that can be used to probe and resolve secondary nucleation events on the surface of primary fibrils.

To address these issues, several experimental efforts have been undertaken to identify nucleation sites on Aβ-42 fibrils (*15*), develop antibody-based scanning methods to selectively investigate primary and secondary nucleation pathways (*17*), and use high-throughput kinetic aggregation assays to capture secondary nucleation events associated with Aβ-42 protein isoforms (*16*, *20*) and tau 304-380 protein fragments (*30*). In addition, fluorescence microscopy has been demonstrated to provide valuable information on the early stages of oligomer adsorption processes on Aβ-42 fibril surface (*31*, *32*), and electron microscopy has been used to determine the endpoints of secondary nucleation by recording the onset of mature fibrils (*33*). In general, experimental work to date has relied mainly on using fluorophores (e.g., Alexa and thioflavin-S) covalently bound to protein aggregates to probe the various stages of secondary nucleation. These studies (*15*, *30*, *33*–*37*) have substantially increased our understanding of oligomer-fibril interfacial interactions. However, it warrants consideration that the attachment of extrinsic fluorophores to small Aβ oligomers could influence particle size (*38*), affect fibril structure (*39*), and perturb the native functionality of proteins. Furthermore, using multiple labels in the context of secondary nucleation can also artificially constrain the conformation and alter the dynamics of oligomers on primary fibrils.

As protein aggregation mostly occurs in solution, it would be desirable to directly resolve fibril surface-driven secondary nucleation events in a label-free manner at nanometer length scales while maintaining the proteins in a hydrated medium. AFM capable of profiling hydrated interfaces is one such surface-sensitive technique that can meet the demands specified above. Building on the observations from previous secondary nucleation studies, we postulated that it should be feasible with a high-resolution AFM to record elementary information such as oligomer size, shape, and adsorption site on individual fibrils with sub-10-nm diameter. Operating the AFM in an aqueous solution medium would allow the diffusion of oligomers on the fibril surface. To this end, we devised a series of liquid cell-compatible AFM experiments to chronologically (from ~30 s to 250 hours) resolve secondary protein aggregates from oligomers to fibrils generated on the primary Aβ-42 fibril surface spanning the embryonic, growth, and endpoint stages of secondary nucleation on a benign gold surface. The size, shape, morphology, and adsorption profile of the diverse secondary protein aggregates were resolved and quantified from AFM height maps. Our computational models of preformed secondary oligomer orientations on double-layered fibril surfaces from molecular dynamics (MD) simulations support AFM observations that the small oligomer binds stably at the fibril edge. Combining label-free imaging experiments with simulations we provide practical insights into the early stages of Aβ-42 oligomer fibril interfacial interactions.

## RESULTS

### Characterization of Aβ-42 primary fibrils as the seeding layer

**Fig. 2.**
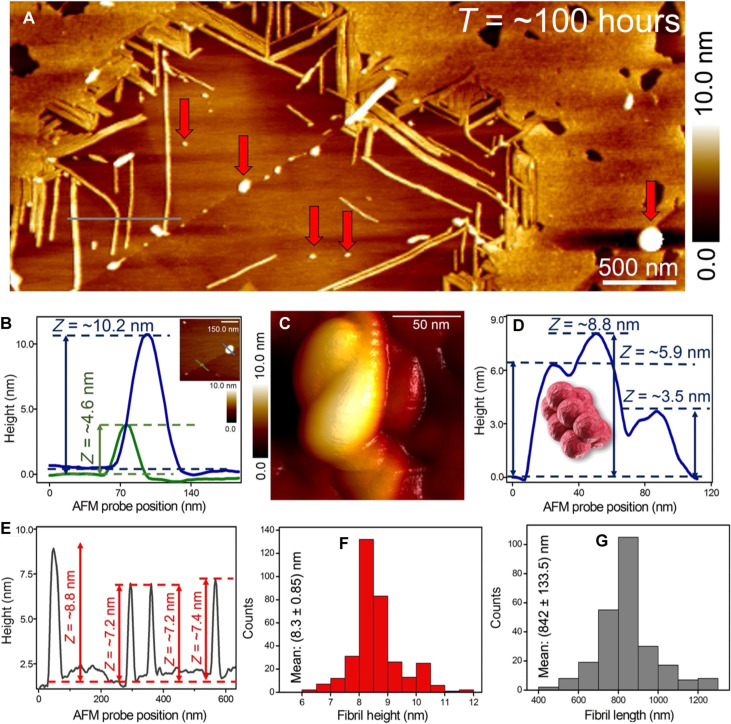
Characterization of primary Aβ-42 fibril morphology. (**A**) Large-area AFM image showing submonolayer coverage of Aβ-42 fibrils. The red arrow indicates that the mature fibrils are also present together with oligomers of varying sizes. (**B**) Cross-sectional height profiles (blue and green traces) extracted along individual Aβ-42 oligomeric particles (indicated by corresponding blue and green lines) are shown in the AFM image (B, inset). (**C**) A representative spatially well-resolved AFM topograph of a single Aβ-42 oligomer. (**D**) Height profiles extracted along multiple points of the Aβ-42 oligomer shown in the AFM image (C) confirm the local height differences within a single oligomeric particle. (**E**) The height profile of single fibrils measured along the gray line indicated in the AFM image shown in (A). On the basis of the quantitative analysis of the AFM images of Aβ-42 fibrils incubated for ~150 hours, we extract a mean fibril height of (8.3 ± 0.85) nm (**F**, red histogram) (*n* = 340) and mean fibril length of (842 ± 133.5) nm (**G**, gray histogram) (*n* = 251).

We start with the morphological characterization of the seeding layer. Aβ-42 solution was prepared using similar protocols and identical peptide concentration (see Materials and Methods for further details on amyloid solution synthesis) as described in our previous work (*26*). Figure 2A is a large-area AFM image showing predominantly mature Aβ-42 fibrils formed after ~100 hours of incubation of Aβ-42 monomer solution at room temperature and then deposited on the gold surface. The AFM image was recorded after the deposition of 20 μl of Aβ-42 solution [phosphate-buffered saline (PBS) solution (pH 7.4)] on the gold substrate followed by removing the PBS solution after ~10 min with water injected through the liquid-cell port. The Aβ-42 fibrils appear to be arranged neatly (Fig. 2A) in a submonolayer coverage on the gold surface. In addition, to the elongated fibrils, the presence of particles of varying sizes is also visible in the AFM topograph (indicated by red arrows in Fig. 2A), which could represent the oligomers present along the Aβ-42 primary aggregation pathway. To quantify oligomer height, we measured the cross-sectional profile (indicated by blue and green lines in height traces in Fig. 2B) across the particles shown in the AFM image in the right corner inset of Fig. 2B. The height profiles clearly show the differences in the size of the particles adsorbed directly on the gold surface. Closer examination of the resolved larger particle reveals local morphological variations (Fig. 2C) that are then quantified using height profiles extracted along the oligomers as shown in the trace plot in Fig. 2D. Regions without any surface coverage of protein aggregates are also seen in the AFM topograph (Fig. 2A), which is a useful baseline to precisely measure the actual height of both oligomers and fibrils. Figure 2E is a cross-sectional profile extracted along the gray line marked across single fibrils as shown in Fig. 2A.

**Fig. 3.**
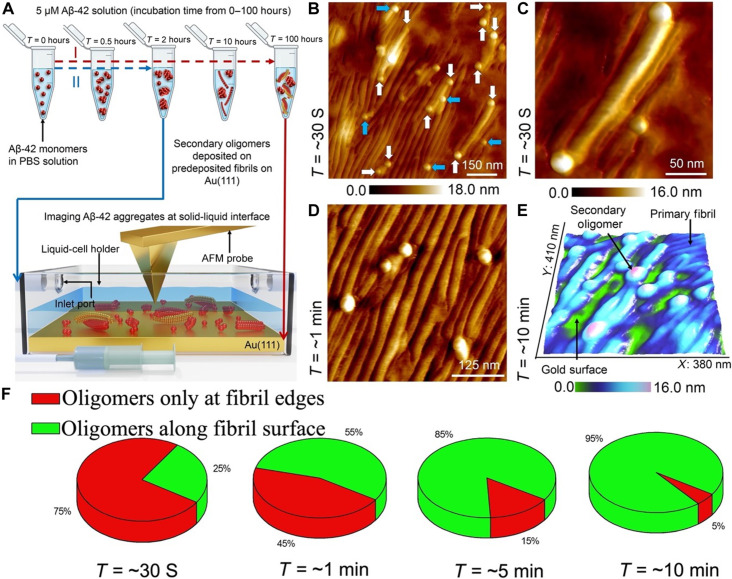
Capturing early stages of oligomer adsorption on Aβ-42 fibrils. (**A**) Diagram of Aβ-42 monomer solution incubation and aggregation process through primary nucleation pathway (highlighted in red dashed arrow) and secondary nucleation pathway (highlighted in blue dashed arrow) studied using liquid-cell setup (shown in the bottom section). Objects in both schematics are not shown to scale. (**B**) AFM topographic image of secondary oligomers adsorbed on the edges (indicated by white arrows) and along the surface (indicated by blue arrows) of Aβ-42 fibrils predeposited on gold substrate. (**C**) High-resolution AFM image of secondary oligomers adsorbed at the edges of a primary fibril. (**D**) AFM image showing several secondary oligomers adsorbed along the surface of primary Aβ-42 fibril surface. (**E**) Three-dimensionally represented AFM image of secondary oligomers adsorbed along the secondary fibril surface. The size of an oligomer is calculated on the basis of the total height measured on top of the oligomer adsorbed on the primary fibril and by subtracting the fibril height with respect to the underlying bare gold surface. AFM images shown in (B), (D), and (E) were recorded after ~30 s, ~1 min, and ~10 min of depositing the secondary oligomer solution onto the predeposited Aβ-42 fibrils. (**F**) Pie chart normalized distribution (over an area of 500 nm^2^) based on AFM images of secondary oligomers adsorbed only at the edges and along the surface of Aβ-42 fibrils recorded at periods of ~30, 60, 300, and 600 s, respectively.

The qualitative AFM data (Fig. 2A) and sectional analysis (Fig. 2E) indicate that fibril height does not vary substantially within the template layer. To obtain the diameter of a single oligomer or a fibril on a flat surface, we calculate the average particle height (from ~3 to 5 sectional profiles per particle) and use the average height value to estimate diameter (height equals diameter in a sphere). We rely on particle height from AFM topographs as this value is independent of AFM tip geometry. From numerous large-area AFM images recorded at different regions of the sample and on the basis of height profile analysis conducted on several hundred single fibrils, we extract a mean diameter of (8.3 ± 0.85) nm (red bar histogram Fig. 2F) and mean length of (842 ± 133.5) nm (gray bar histogram Fig. 2G). The coexistence of a small population of oligomers even at the saturation phase of amyloid aggregation and morphology of mature fibrils is consistent with our previous reports (*26*) and with independent profiling studies of surface-confined Aβ-42 aggregates (*28*, *40*, *41*). The predominant presence of elongated fibrils arranged in a close-packed manner with near-homogeneous diameter and their ability to remain intact even after the in situ exchange of liquids confirmed that the primary Aβ-42 fibril–composed layer could be well suited to serve as a template layer to study the secondary nucleation process. Consequently, Aβ-42 fibrils formed after ~100 hours of incubation of monomeric peptides and then deposited on a gold surface were regularly used throughout this study as the seeding layer on which secondary nucleation events were visualized using liquid-based AFM (as shown in the schematic in Fig. 3A).

### Secondary oligomers prefer to adsorb initially on Aβ-42 fibril edges

Several lines of evidence indicate that monomers and oligomers adsorb on primary Aβ-42 fibrils surface freely (*15*, *16*, *31*, *32*). The AFM images we recorded during the embryonic stages of secondary nucleation indicated that oligomers (formed after incubating Aβ-42 monomer solution for a period of ~2 hours) exhibited a propensity to initially adsorb on the edges of primary fibrils. Our AFM setup requires at least ~15 to 30 s for the AFM probe to approach the surface followed by establishing stable AFM imaging. Consequently, it has not been feasible to capture the secondary nucleation events within a 0- to 30-s time window due to instrumental limitations. Note that time point 0 refers to the period at which secondary oligomers (volume: 50 μl) were deposited into the liquid-cell setup. Figure 3B is an AFM image captured at a time point of ~30 s after depositing secondary oligomers on the preformed Aβ-42 fibrils template on the gold surface. On first inspection, it is evident that a stable set of oligomers have adsorbed on the primary Aβ-42 fibrils. A closer examination of the AFM topograph reveals that oligomers are mainly adsorbed on fibril edges (indicated by the white arrows in Fig. 3B), and only a small population of oligomers are adsorbed on the fibril backbone (indicated by blue arrows in Fig. 3B). The adsorption profile of the oligomers at fibril edges is resolved from the AFM topograph (Fig. 3C), where individual oligomers are observed to be positioned at the fibril edges.

Figure 3D is an AFM image recorded after a period of ~1 min showing oligomers adsorbed on the backbone of closely packed primary fibrils. To better visualize the adsorption profile of the individual oligomers along the fibril backbone, height maps were analyzed in three-dimensional (3D) format. Figure 3E is a 3D rendered AFM height map showing that primary fibrils are structurally intact even after oligomer adsorption. To further test for initial preferential adsorption of the secondary oligomers on the edges of primary fibrils, we repeated the experiments under identical conditions in two separate trials in a buffer salt solution medium. Figure 3F is a pie chart distribution (normalized over an area of 500-nm^2^ AFM images) recorded over independent trials acquired from ~0.5 to 10 min showing the distribution of adsorption sites of secondary oligomers on primary Aβ-42 fibrils. The population of oligomers adsorbed on edges are coded in red (geometry as shown in Fig. 3C), and the oligomers adsorbed on the backbone of fibrils are coded in green. Although Aβ-42 oligomers adsorbed on primary fibrils were observed to proliferate on the primary fibril surface from the time-elapsed AFM images, the Aβ-42 oligomers adsorbed directly on the gold surface did not show any tendency for on-surface aggregation, possibly due to the stronger interactions with the underlying gold surface. The AFM findings recorded at the solid-liquid interface indicate that secondary oligomers initially adsorb on fibril edges first. Then, over time, oligomers also start to adsorb at multiple sites along the fibril backbone.

### Quantifying Aβ-42 oligomer-fibril surface interactions via molecular simulations

**Fig. 4.**
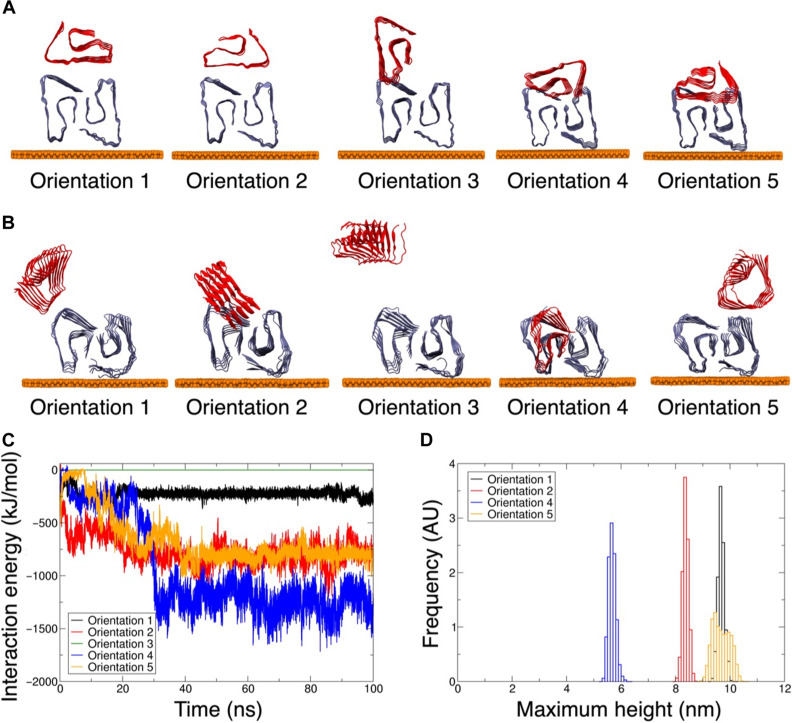
MD simulations of Aβ-42 oligomers adsorbed along the backbone and at the edge of the Aβ-42 fibril. (**A**) Initial orientations showing side-on views of the oligomer (colored red) adsorbed in various starting configurations on the fibril template (colored blue). The oligomer is initially placed either along the primary fibril surface (orientations 1 and 2) or at the fibril edge (orientations 3, 4, and 5) (**B**) Final structures formed following 100 ns of MD. See fig. S1 for plan views of the complexes from above. (**C**) Comparison of oligomer-fibril total interaction energies as estimates of the binding strength of each complex. (**D**) Distribution of secondary oligomer height profiles above the gold platform, comparing different morphologies assembled in the four calculated successful oligomer-fibril adsorption profiles (orientation 3 is omitted as the oligomer dissociated from the fibril surface). AU, arbitrary units.

To shed light on the thermodynamic factors driving oligomer-fibril orientations of the secondary oligomer binding to edges and along the backbone of primary Aβ-42 fibrils, we modeled the secondary oligomer-fibril interface using MD computer simulations. Previous reports suggest that dodecamers (12-mers) of Aβ-42 may seed the formation of extended, linear-fibrillar β sheet structures (*42*) and may be the smallest long-lived soluble toxic oligomer for Aβ42 forming fibrillar structures (*43*). We used the dodecamer model (see Materials and Methods) as a primary deposited fibrillar layer on the hydrated gold substrate with the secondary oligomer placed initially either directly above or on top of the fibril surface (orientations 1 and 2) or at the edge of the primary fibril (orientations 3, 4, and 5) as shown in the initial structures in Fig. 4 (A and B). The extensive large-area atomic-resolution MD simulations identify the most stable oligomer-fibril interface during the initial assembly at experimentally inaccessible submicrosecond timescales.

The MD simulations starting from orientation 1 along the fibril surface show only weak oligomer—fibril interactions with the hexameric peptide oligomer loosely associating with the surface of the dodecameric fibril unit and remaining in that orientation during 0.1 ms of free dynamics in the room temperature, fully solvated environment (Fig. 4B). By contrast, in simulations starting from orientation 2, the oligomer migrates to make a strong contact close to the fibril edge after 100-ns dynamics. In one of the starting orientations (orientation 3) where the oligomer is initially placed at the edge of the fibril, the peptide leaves the surface of the fibril immediately and does not rebind during the simulations, while starting structures in orientations 4 and 5 also form stable oligomer-fibril complexes. Orientation 4 leads to the most stable complex (Fig. 4B) with the oligomer added directly in front creating a growing chain of fibrils in one layer. In orientation 5, the oligomer moves to the top of the fibril by the end of 100 ns of dynamics (Fig. 4B) to create an intermediate strength complex near-isoenergetic with the complex assembled from starting orientation 2 (Fig. 4C). The principal Coloumbic interactions between complementary charged and polar residues that drive the formation of the tight oligomer-fibril interface are highlighted in fig. S1 together with the minor contribution from steric van der Waals contacts between nonpolar sites.

**Fig. 5.**
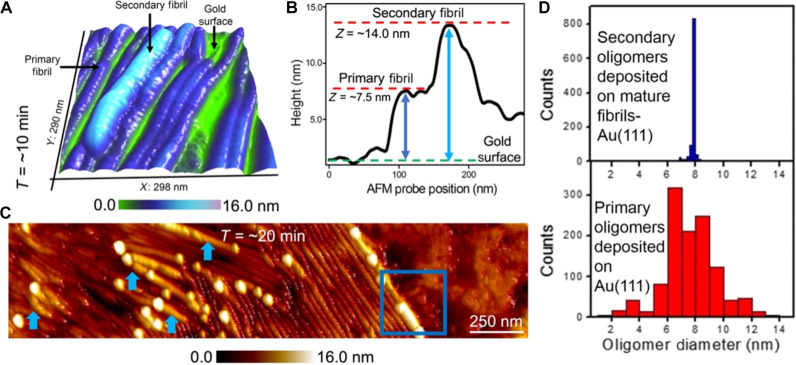
Quantifying secondary fibril and oligomer size. (**A**) Three-dimensionally represented AFM image recorded at a period of ~10 min after deposition of the primary oligomers on preformed Aβ-42 fibrils. (**B**) The cross-sectional profile extracted along the primary and secondary fibrils in AFM image shown in (A). (**C**) Time-elapsed AFM image recorded ~20 min after initiating the secondary nucleation process showing secondary oligomers to be adsorbed mostly along the primary and secondary (indicated by blue arrows) Aβ-42 fibrils. (**D**) On the basis of the height traces measured on secondary Aβ-42 oligomers adsorbed on fibrils (top blue bar histogram, *n*: 1066) and directly on the gold surface (bottom red bar histogram, *n*: 1168), we extract mean oligomer diameters of (7.9 ± 0.2) nm and (7.3 ± 1.9) nm, respectively. The Aβ-42 oligomers in both experiments were formed after the Aβ-42 monomer solution was incubated for ~2 hours.

We then computed the distribution of maximum heights for each orientation on the surface to benchmark the oligomer—fibril height profiles sampled in the molecular simulations against the particle height distribution profile measured using AFM. Our MD data predict that all starting orientations equilibrate to form a complex with steady, plateaued heights during the MD simulations (fig. S1F). Orientations 1 and 5 samples have the same tall height profiles (~9.5 nm) with the secondary oligomer bound on top of the layer. Orientation 4 samples the shortest height due to the oligomer side-on addition to the growing fibril chain, and orientation 2 shows intermediate height profiles with oligomer at the edge of fibrils. The distribution of model oligomer height profiles during the final 50 ns of dynamics monitors the secondary oligomer adsorption along the backbone and at the edges of Aβ-42 fibrils. Our data reveal that our model orientation 2 which adopts a final orientation at the edge of the fibrils (Fig. 4B) with a mean height of (8.3 ± 0.6) nm (Fig. 4D) is a good match to the upper limit of the mean secondary oligomer height of (7.9 ± 0.2) nm obtained from AFM experiments (see Fig. 5D, discussed later). Note that the MD heights are the heights of the full oligomer-fibril complex above the gold platform, while the AFM-measured heights of the secondary oligomer are heights above the fibrillar layer, and so the MD model of the early stage and small oligomer-fibril complex may provide a representative structure and morphology of the secondary oligomers detected by AFM. The computed oligomer-fibril binding interface (Fig. 4B) thus provides an atomic-resolution model of the initial complex formed at submicrosecond timescales that seeds the secondary nucleation events at the early stages of fibril surface catalyzed self-assembly of oligomers (in this case, a hexamer). At the much longer experimental timescales after the first ~30 of depositing the secondary oligomer solution onto the predeposited Aβ-42 fibrils, most oligomers in the experiments are seen to be adsorbed at the edge of fibrils (see Fig. 3F), as opposed to the fibril backbone. The corresponding model of the edge-on bound oligomer-fibril complex (orientation 2) is predicted to be thermodynamically stable, intermediate in energy between the loosely bound, weakly associated interface (orientation 1; Fig. 4C) and the most stable, partially buried oligomer binding site (orientation 4). This most stable small, 5- to 6-nm seed with the oligomer added directly in front creating a growing chain of fibrils in one layer is consistent with the formation of an energetically favorable but highly active seed that rapidly grows to form a tightly packed fibrillar layer. Hence, MD simulations provide a range of particle sizes and shapes that illustrate the atomic-scale structure, dynamics, and energetics of both the edge-on binding interface and the packing of the fibril.

### Monitoring evolution in size and shape of secondary Aβ-42 oligomers and fibrils

Time-lapse AFM imaging revealed that past the embryonic stages and the initial preference for edge-on binding to the Aβ-42 fibrils, the oligomers from the solution phase adsorbs indiscriminately on the Aβ-42 fibrils eventually forming lengthy fibrils. Aβ-42 fibrillar aggregates are known to form in solution after several hours of aggregation along the primary nucleation pathway (*26*). Under identical experimental conditions, we noticed that secondary fibrils appeared within several minutes after the nucleation process was triggered on the primary Aβ-42 fibril surface. Figure 5A is a 3D AFM topograph revealing the presence of a secondary fibrillar aggregate positioned on top of a primary fibril. This specific AFM data were captured within ~10 min of starting along the secondary nucleation process. In all the trials conducted as part of the study, the first onset of secondary fibrils was observed to occur within minutes of depositing the oligomers on the primary Aβ-42 fibril template. This is in agreement with kinetic assay–based studies showing that protein aggregates are formed in an accelerated manner during secondary nucleation in comparison to the primary nucleation process (*16*, *19*), a feature that has been attributed to the properties of the primary fibril surface that provides catalytic centers for nucleation and elongation (as characterized in detail below).

To quantify the heterogeneous morphology of the secondary fibrils, we measured fibril length and diameter. The length of the secondary fibrils can be directly measured from the AFM topographs using point-to-point measurement markers in the analysis software. To obtain the secondary fibril height adsorbed on a primary fibril, it is important to select a region on the sample as shown in Fig. 5A where the primary and secondary fibrils are discernible together with the bare gold surface within a single image frame. Thereafter, we extract the primary fibril height through a cross-sectional profile analysis as shown in Fig. 5B. The height of the primary fibril is then subtracted from the total height measured to yield the secondary fibril height with respect to the underlying gold surface. As height equals the diameter of cylindrical objects, we use the fibril height to obtain the fibril diameter. The same procedure was also used to extract the height of the spherical-shaped secondary oligomers, from which we obtained the oligomer diameter. On the basis of cross-sectional profile analysis (Fig. 5B) on 185 secondary fibrils (resolved at a time point of ~10 min), we measure a mean fibril length of (250 ± 55 nm) and a mean fibril diameter of (6.5 ± 0.2) nm. Figure 5C is a representative large-area AFM image recorded at a time point of ~20 min showing the evolution in size and shape of the newly formed protein aggregates. It was at this time point that we observed a distinct increase in the size of the secondary oligomers and an increase in the prevalence of secondary fibrils (marked with blue arrows in Fig. 5C). The AFM data also show the secondary oligomers assembled in a more closely packed structure (indicated by the blue box in Fig. 5C), which could be kinetic intermediates on the path to elongated fibrils. On the basis of sectional analysis of secondary oligomers detected at a time point of ~20 min, we calculate a mean oligomer diameter of (11.9 ± 0.3) nm for that stage of growth. This measurement quantifies the gradual increase in secondary oligomer size over time as we obtained a mean secondary oligomer diameter of (7.9 ± 0.2) nm based on AFM images recorded at a time point of ~30 s (top blue histogram, Fig. 5D). This growth stands in stark contrast to the much broader size distribution of primary oligomers incubated for the same period of ~2 hours and then deposited on a gold surface, showing a mean oligomer diameter of (7.3 ± 1.9) nm (bottom red histogram, Fig. 5D). Comparing the differences in oligomer size distributions incubated under identical conditions but only differing in the choice of surface on which they were deposited (secondary nucleation: Aβ-42 fibril template layer or primary nucleation: gold surface) suggests that Aβ-42 fibril surface appears to screen the population of oligomers and selectively adsorb only a distinct class of oligomers. Previous, kinetic studies have indicated that the surface of amyloid fibrils can stabilize specific oligomeric forms (*44*). The findings from our work using AFM further highlight the role of the Aβ-42 fibril surface in mediating and directing the oligomer adsorption and assembly process.

### A few but not all primary Aβ-42 fibrils are highly catalytic

**Fig. 6.**
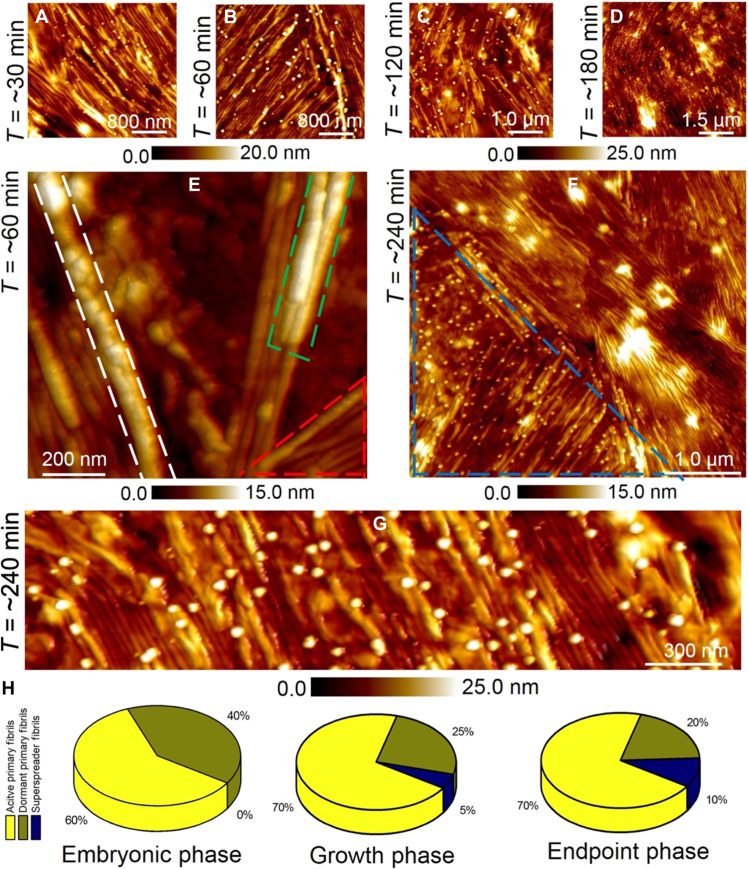
Identifying differences in catalytic activity of fibrils. (**A** to **D**) Large-area AFM images were recorded at ~30, 60, 120, and 180 min after deposition of the oligomers on Aβ-42 primary fibrils. (**E**) High-resolution AFM image (recorded at the time point of ~60 min) showing fibrils exhibiting high catalytic activity (indicated in white dashed and green dashed regions). The red dashed region in (E) highlights primary fibrils with no adsorbed oligomers. (**F**) Large-area AFM image recorded at a time point of ~240 min showing regions with high fibril catalytic activity (indicated by blue dashed region). The morphology of the secondary oligomers and fibrils did not vary beyond this time point indicative of the endpoint of the secondary nucleation pathway. (**G**) High-resolution AFM image recorded at the time point of ~240 min showing local differences in catalytic activity. (**H**) Pie chart showing the normalized distribution (over an area of 500 nm^2^) of active primary fibrils (coded in yellow), dormant primary fibrils (coded in dark green), and superspreader primary fibrils (coded in blue), based on AFM images recorded during embryonic, growth, and endpoint phases of Aβ-42 secondary nucleation pathway.

Next, we present the results from a time series of AFM measurements conducted during the growth phase out to the endpoint stages of secondary nucleation. Figure 6 (A to D) shows large-area AFM images recorded at time points of ~30, 60, 120, and 180 min, respectively. Oligomeric particles are visible together with elongated secondary fibrils in all the AFM images during the growth and endpoint stages. From the AFM data, we did not observe any substantial increase in the diameter of secondary fibrils during the growth phase (*t*:~30 to 180 min, fibril diameter: 6.0 to 7.8 nm) when compared to the diameter of secondary fibrils resolved during the embryonic stages (*t*: ~10 to 30 min, fibril diameter: 6.0 to 9.5 nm). This information suggests that secondary fibrils could adopt the morphology of the preformed primary fibrils templates which we calculated to have a mean diameter of (8.3 ± 0.85) nm (Fig. 2F). In stark contrast to the fibril diameter property, the length of secondary fibrils was observed to greatly increase during the growth phase of secondary nucleation. For example, at a time point of ~180 min, we measured a mean length of (2200 ± 145) nm during the growth phase for secondary fibrils, whereas the mean length of fibrils detected at a time point of ~10 min was calculated to be (250 ± 55) nm during the embryonic phase. After ~220 min, the fibril length was observed to reach a saturation level indicative of endpoint stages of secondary nucleation.

When analyzing the full library of AFM data on primary and secondary fibrils recorded during embryonic, growth, and endpoint stages, we found that the vast majority of primary fibrils were catalytically active. The remaining population remained dormant throughout the secondary nucleation process. In addition, we detected a relatively small number of primary fibrils that were extremely catalytically active, which may have a disproportionate influence on the growth kinetics. Such catalytically hyperactive fibrils were previously reported for Aβ-42 protein aggregates resolved using fluorescence microscopy and classified as superspreaders (*31*). Figure 6E is a spatially well-resolved AFM image recorded at a time of ~60 min that reveals the details of local differences in the catalytic activity of neighboring fibrils. The fibril indicated by the white dashed line clearly shows the secondary oligomers arranged in a dense configuration, mostly likely kinetic intermediates formed before the generation of a secondary fibril. In contrast, the green dashed line in the same frame shows mature secondary fibrils growing on top of each other and primary fibrils with no catalytic activity (red dashed area). In addition to the existence of catalytically dormant primary fibrils detected during endpoints of secondary nucleation, we also observed small oligomers to be present at the endpoint stages. Fig. 6F is a large-area AFM image recorded at the endpoint stages of secondary nucleation (*t*: ~240 min) showing predominantly mature and elongated fibrils. Notably, small oligomeric particles were still detected in the AFM image (within the blue dashed region) confirming that not all secondary oligomers undergo conversion to form insoluble fibrils (Fig. 6F). A spatially well-resolved AFM image (Fig. 6G) recorded at the time point of ~240 min shows the individual oligomers adsorbed on the primary Aβ-42 fibrils and the presence of a dense population of secondary fibrils. Figure 6H is a series of pie chart plots mapping the distribution of active primary fibrils, dormant primary fibrils, and superspreader primary fibrils based on AFM data recorded during the embryonic (*t*: ~0.5 to 10 min), growth (*t*: ~10 to 120 min), and endpoint (*t*: ~120 to 240 min) phase of secondary nucleation. As the primary fibrils (active, dormant, and superspreaders) are morphologically identical at the resolution of the AFM, we propose that variations in chemical texture such as hydrophobic patches (Val^18^ + Ala^21^ and Val^40^ + Ala^42^) known to be present along the surface of Aβ-42 fibrils (*34*, *45*) may influence the degree of catalytic activity of the primary fibrils. The structural heterogeneity of near-isoenergetic oligomer-fibril complexes predicted from the MD simulations is also consistent with a heterogeneous population of fibrils varying according to their catalytic activity that may depend on which fibril patches are surface-exposed adsorption sites, which in turn seeds the assembly and growth process.

### Surface roughness as a parameter to fingerprint fibril subpopulations

**Fig. 7.**
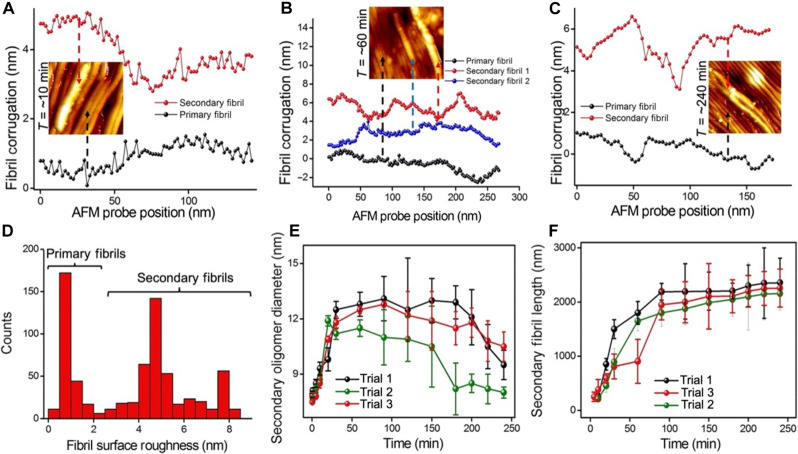
Profiling corrugations along primary and secondary fibrils. (**A** to **C**) The plot of surface corrugation measured along the backbone of secondary and primary fibrils resolved in the inset AFM images recorded at multiple time points of ~10, 60, and 240 min, respectively. (**D**) Plot showing the distribution of average fibril surface roughness measured along the length of the fibrils resolved from the AFM height maps. The lower surface roughness values ranging from ~0.4 to 2.2 nm correspond to the roughness measured along the primary fibrils, and the higher values correspond to the secondary fibrils. (**E**) Plot showing the diameter of secondary Aβ-42 oligomers measured on Aβ-42 primary fibrils based on AFM data collected at multiple time points from ~0.5 to 250 min. Particle diameter is calculated from AFM height profile analysis of individual oligomers detected both on the edges and along the surface of Aβ-42 fibrils. (**F**) Plot monitoring the evolution of secondary fibril length over time (~10 to 250 min) obtained from AFM height maps. The individual data points shown in (E) and (F) in the black plot (trial 1) and red plot (trial 3) are based on AFM measurements conducted in buffer salt solutions. The green plots in (E) and (F) are based on AFM measurements (trial 2) conducted in a water medium. Data shown in (E) and (F) are expressed as mean ± SD.

Mature Aβ-42 fibrils are known to be chemically heterogeneous. We asked whether nanoscopic profiles extracted on primary and secondary fibril surfaces could reveal different surface textures, and if so could this information be used to differentiate between fibril subpopulations? To address this, we carefully traced the corrugations along the surface of both primary and secondary fibrils. Figure 7A shows the comparative line scan extracted along the primary fibrils (black trace) and secondary fibrils (red trace) as resolved in the AFM image recorded at a time point of ~10 min. The local differences in corrugations are highlighted from the AFM traces, wherein the secondary fibrils appear to have higher corrugation than the primary fibrils. The average roughness value can then be determined by computing the root mean square (*R*q) values using the functions available in the commercially available software (Nanoscope, Bruker) along several areas of a single fibril. To verify if this trend holds for primary and secondary fibrils recorded at multiple time points of secondary nucleation, we analyzed the surface corrugation of several hundreds of fibrils resolved from the AFM measurements. Figure 7B is another representative surface corrugation analysis conducted on primary fibrils (black trace) and secondary fibrils (red and blue traces) resolved within a single AFM image (top inset of Fig. 7B) captured at a time point of ~60 min, confirming that primary fibrils are less corrugated than secondary fibrils. This trend was also observed to hold at endpoint stages of secondary nucleation (Fig. 7C) where the primary fibril surface (black trace) is less corrugated than the secondary fibril (red trace) as resolved from the corresponding AFM image (inset of Fig. 7C) recorded at the time point of ~240 min. On the basis of the set of trace plots (Fig. 7, A to C) recorded at different time points of the secondary nucleation process, we map the average surface roughness of the fibrils (Fig. 7D). Primary fibrils were calculated to have a surface roughness value ranging from ~0.4 to 2.2 nm, and the secondary fibrils were observed to display higher surface roughness values ranging from ~2.5 to 8.5 nm. The secondary fibrils with higher surface corrugation were mainly generated during the growth phase (*t*: ~10 to 120 min) of secondary nucleation.

Next, we summarize the evolution in secondary oligomers and fibrils size-resolved in our study. Figure 7E is a plot showing the changes in secondary oligomer size measured from a time point series of ~0.5 to 250 min obtained from three different trials. The plot shows that the size of the secondary oligomers steadily increases from the growth to the lag phase but decreases during the endpoint phase. In the case of secondary fibril morphology changes, the diameter of secondary fibrils did not vary substantially when measured between embryonic and endpoint phases, but the length was observed to increase over time. Figure 7F is a plot showing the evolution in secondary fibril length based on AFM topographs recorded from ~5 to 250 min (as fibril onset was not spontaneous) over three independent trials. Note that the experimental conditions used in the three trials were almost identical except for that in trial 1 (black trace, Fig. 7, E and D) and trial 3 (red trace, Fig. 7, E and D) AFM imaging was conducted in buffer salt solution conditions, while trial 2 (green trace, Fig. 7, E and D) was conducted in a pure water environment.

## DISCUSSION

Transient Aβ-42 oligomeric assemblies generated on preformed fibrils could be highly toxic. Yet, it is not trivial to capture and characterize the transient oligomeric assemblies on individual fibril surfaces. We experimentally resolved and quantified the heterogeneous size distribution of secondary oligomers and fibrils on Aβ-42 primary fibrils using liquid-based AFM. The AFM topographs recorded during the embryonic stages of secondary nucleation reveal that the edges of primary fibrils act as the preferential binding sites for the secondary oligomers. The size distribution of secondary oligomers on primary fibrils was observed to be narrower (7.9 ± 0.2) nm when compared to oligomers incubated for identical periods and later deposited on the gold surface (7.3 ± 1.9) nm, suggesting that primary Aβ-42 fibrils could potentially be screening oligomers of specific size ranges. The spacing between the secondary oligomers ranged from ~20 to ~100 nm on the primary fibril backbone. On the basis of the AFM measurements, we did not find any indication for either short or long-range periodicity of the catalytic sites along the primary Aβ-42 fibril surface. Next, AFM data recorded during growth and endpoint stages of secondary nucleation confirmed the presence of a small but stable population of primary fibrils, which displayed higher catalytic activity compared to their adjacent counterparts. In general, the key findings reported in this study using AFM highlight the heterogeneous chemical nature of Aβ-42 fibril surface, which is in agreement with experimental results obtained using fluorophore-based imaging or kinetic experiments (*30*, *31*, *37*, *46*, *47*). Furthermore, the gradual increase of secondary oligomer size from the growth phase to the lag phase and the subsequent reduction in their size during endpoint stages observed from AFM measurements (Fig. 7E) could be attributed to the recent findings that Aβ-42 fibril surface could also dissociate secondary oligomers (*35*). We anticipate that further detailed morphological and chemical characterization of secondary oligomer interfacial interactions on fibril surfaces using complementary label-free techniques will allow the clarification of chemical drivers governing nucleation and growth of secondary protein aggregates. For example, the structure of the secondary oligomer complex adsorbed on primary fibril edges can be resolved at the atomic scale using graphene-liquid cell-based electron microscopy (*36*), and video-rate AFM (*28*) is well suited for capturing in real-time the dissociation dynamics of secondary oligomers. Furthermore, when nanoscale imaging capability is combined with chemical analytics such as tip-enhanced Raman spectroscopy (*37*), it can register the local chemical structure of the secondary protein aggregates.

Modeling provides additional details on secondary oligomer adsorption and dynamics on primary fibril surfaces at experimentally inaccessible timescales. A previous report on modeling secondary nucleation through coarse-grained MD simulations tracked the association of fragments of Aβ peptides such as Aβ9-40 monomers and dimers along the sides of Aβ-40 fibrils, and it was proposed that the entropic gain upon release of hydration water molecules was a major driving force for the secondary association of monomers (*46*). Our atomistic MD simulations show a similar balance between peptide-peptide and peptide-water interactions in determining the electrostatic stability of the secondary oligomers [see fig. S1 (B to E)]. Discrete MD simulations together with experiments showed that secondary nucleation is the dominant process in Aβ-40 fibril formation kinetics during coassembly with Aβ16-22 (*48*). A more recent study with unbiased MD simulations showed that amyloid peptides first dock onto the fibril tip directly from bulk solution or after binding onto the fibril surface, followed by a substantial population of the fibril tip with peptides, identifying a dock-and-lock mechanism of secondary nucleation (*49*). In this work, our MD models of different starting secondary oligomer orientations on preformed two-layered fibril surface predict that the oligomer binds stably at the fibril edge with a frequently sampled height of 8.3 nm. This value is close to the mean secondary oligomer height of 7.9 nm obtained from the AFM experiments and so provides an atomically resolved model of the early stages of secondary oligomer assembly on primary fibrils of Aβ-42 driven majorly by favorable oligomer—fibril electrostatic interactions. Further, repeat MD simulations with an alternative model of double-horseshoe-like cross–β sheet morphology of Aβ-42 (*45*) support the model prediction that the oligomer binds preferentially at the edge of fibril as shown in fig. S2.

In summary, this paper demonstrates the characterization of Aβ-42 protein aggregates autocatalytically generated on the surface of fibrils using an atomic force microscope. The imaging methodology described here does not require external fluorophores and can be conducted in physiological conditions thus retaining the proteins in a hydrated state. The morphological changes undergone by the oligomers and fibrils formed in an accelerated manner through multiple aggregation steps have been quantified at the single-particle level. Collectively, the knowledge gained from this study may prove to be useful in identifying valid therapeutic targets and explaining how amyloids grow and spread in the AD brain.

## MATERIALS AND METHODS

### Preparation of Aβ-42 solution

High-performance liquid chromatography purified Aβ-42 peptides were purchased from Sigma-Aldrich in lyophilized form. Aβ-42 peptides (0.5 mg/ml) were reconstituted in 10% NH_4_OH. The Aβ-42 solution was agitated for ~30 min at room temperature and stored in individual aliquots (0.5 ml, in Eppendorf protein low-bind tubes). Next, the samples were lyophilized and stored at −20°C until use. Each aliquot was next dissolved in 60 mM NaOH and mechanically agitated for ~30 min to reach a peptide concentration of 100 μM. Aβ-42 concentration was determined by absorbance of stock solutions at 280 nm (ε = 1490 M^−1^ cm^−1^). Next, PBS solution (pH 7.4) from Sigma-Aldrich was added to the Aβ-42 peptides to yield a concentration of 5 μM and obtain a neutral pH value to start the amyloid aggregation process. The Aβ-42 peptides in PBS stock solution were then incubated at room temperature for ~100 hours to allow spontaneous protein assembly.

### AFM measurements in aqueous medium

AFM measurements were conducted using Bruker Multimode 8 AFM with a closed liquid cell at room temperature. For the AFM probe, a scout 150 HAR silicon AFM tip with a high aspect ratio (gold reflective backside coating, resonant frequency: 150 kHz; spring constant: 18 N/m) was used in tapping mode. Before placing the AFM probe in the tip holder, the cantilever was cleaned with acetone for ~30 s followed by rinsing in isopropanol for ~30 s followed by drying the cantilever with compressed air. This cleaning procedure was conducted to remove any contaminants from storing and shipping the AFM probes in a gel pack. For characterizing protein aggregates formed along the primary pathway using liquid AFM, the following protocol was used; upon inserting the AFM probe in the tip holder, 20 μl of Aβ-42 solution incubated (*t*:~100 hours) at room temperature was deposited on Au(111) substrate placed within the liquid-cell setup. Gold film with 200-nm thickness on mica substrate was obtained from Phasis Inc., Switzerland. The as-received substrates were flame annealed (total exposure time: ~10 s, flame length was maintained at about 2.54 cm 1 inch and moved back and forth gently). The annealed gold thin film on the mica substrate was then cooled for about 10 min before adhering them firmly on metal stubs and positioning them within the liquid-cell holder. This process results in forming gold thin films without pinhole surface defects. On the basis of our previous studies on various solid surfaces (graphene, gold, mica, and Si) (*26*), we do not observe any differences in the selectivity of Aβ-42 mature fibril arrangement on flame-annealed gold. The Aβ-42 solution volume was maintained at 20 μl, and the monomer concentration of the Aβ-42 solution was kept fixed at 5 μM in PBS solution (pH 7.4) for all AFM measurements. Ten minutes after deposition of the Aβ-42 solution on Au(111), the liquid cell was gently flushed with 1 ml of ultrapure water, and AFM imaging was conducted in a water medium to analyze the primary oligomers and fibrils adsorbed on Au(111). The following protocol was used for studying the secondary nucleation events on preformed Aβ-42 fibrils (solution incubation time, ~100 hours). Fifty microlites of Aβ-42 solution incubated for ~2 hours was deposited on Aβ-42 fibrils preadsorbed on Au(111). This incubation period (~2 hours) was found optimal as we were able to resolve a heterogeneous population of protein aggregates formed at this incubation point (Fig. 5D, red histogram) using AFM. Hence, we selected this oligomer-rich solution to deposit on the primary fibrils to initiate the secondary nucleation events. To resolve the secondary nucleation events, AFM imaging was conducted in a PBS solution medium (trials 1 and 3) and also in a clean water (50 μl) medium (trial 2). To avoid tip contamination from the PBS medium, the quality of the tips was constantly monitored, and new tips were exchanged at shorter intervals. The gold substrate and AFM cantilever were fully immersed in the aqueous solution. For image processing, the raw AFM height data were analyzed using Nanoscope analysis (version 1.9, Bruker) software and were only subjected to a flattening procedure before obtaining the height and phase-contrast information.

### MD simulations

The starting models of preformed primary fibrils were obtained from the Aβ-42 dodecamer structure with two symmetric LS-shaped folds of hexamers packed laterally [from the cryo-EM report on the atomic structure of Aβ-42 fibrils (*21*), Protein Data Bank (PDB) code: 5OQV] that we modeled in our previous work (*26*), with the fibril axis parallel to the gold Au(111) surface in a large periodic water-filled box. We then modeled a single layer of LS-shaped hexamer as the secondary oligomer using the same cryo-EM structure of Aβ-42 as different starting orientations—two on top of the double-layered fibril backbone (orientation 1 and orientation 2; see representative conformation in Fig. 3A) and three at the edge (orientation 3, orientation 4, and orientation 5; Fig. 3B) of the primary fibril on gold—water interface (see fig. S1A). In addition to the LS-shaped fibril and oligomer, we also modeled a double-horseshoe-like cross–β sheet morphology of Aβ-42 dodecamer structure having symmetric hexamers stacked laterally (*45*) (PDB code: 2NAO) solved by solution nuclear magnetic resonance with its corresponding hexamer as secondary oligomer. The hexamer was modeled on top or at the edge of the fibril. MD simulations in these seven different starting oligomer-fibril orientations were run using the Gromacs 2018.4 (*50*, *51*) package with the proteins and the gold represented by the CHARMM 36m (*52*) force field and solvated with CHARMM-modified TIP3P (*52*) explicit water molecules. The maximum height profile at the gold-water interface was computed by using Tcl/Tk scripting integrated with Visual Molecular Dynamics (VMD) (*47*). More details of model preparation, MD simulations, and data analyses are given in section S1.

## Supplementary Materials

### This PDF file includes:

Section S1 Figs. S1 and S2
