# Supplementary material for: Accelerated Alzheimer’s Aβ-42 secondary nucleation chronologically visualized on fibril surfaces
Source: Sci Adv. 2024 Oct 25;10(43):eadp5059. doi: 10.1126/sciadv.adp5059 (PMC11506133; doi:10.1126/sciadv.adp5059)
Supplement: Supplementary file 1 — Section S1 Figs. S1 and S2 [file sciadv.adp5059_sm.pdf]

Science Advances
AAAS

Supplementary Materials for

Accelerated Alzheimer's Aβ-42 secondary nucleation chronologically visualized on fibril surfaces

Peter Niraj Nirmalraj *et al.*

Corresponding author: Peter Niraj Nirmalraj, peter.nirmalraj@empa.ch;
Damien Thompson, damien.thompson@ul.ie

Sci. Adv. **10**, eadp5059 (2024)
DOI: 10.1126/sciadv.adp5059

This PDF file includes:

Section S1
Figs. S1 and S2

Supplementary section S1.

S1.1 Preparation of the models. To approximate the experimental atomically flat Au(111) surface, a 15 nm x 15 nm Au(111) two-layer slab with thickness of 0.3 was used. The infinite Au(111) surface was obtained by applying periodic boundary conditions, and the Au atoms were given net zero charges and held fixed in their crystallographic positions throughout the simulations. The Aβ-42 dodecamer (12-mer) structure was used as a primary deposited fibrillar fold with two symmetric LS-shaped folds of hexamers packed laterally, obtained from the cryo–electron microscopy (cryo-EM) decamer (10-mer) structure of the Aβ-42 fibrils (PDB code: 5OQV). The initial structures of pre-formed fibrils were oriented with the fibril axis parallel to the Au(111) surface, with the two LS-shaped folds laterally placed on top of gold. The structures were placed at a starting distance of at least 6 Å above the gold substrate. For the secondary oligomer, we modelled a single layer of LS-shaped hexamer structure of Aβ-42 from the cryo-EM. Five different starting orientations of secondary oligomer on primary fibrils of Aβ-42 were modelled – Orientations 1 and 2 with oligomer on top of the double layered fibril backbone (see representative conformation in Figure 4A and starting conformations in Figures S1A, B) and Orientations 3, 4 and 5 (Figure 4B, and Figures S1A, B) with oligomers at the edge of the primary fibril. For repeat simulations, the structure from PDB code 2NAO was used, sampling hexamer binding on-top (Figure S2A) and at the edge (Figure S2B) of the stacked dodecamer.

S1.2 Molecular dynamics (MD) simulation details

The proteins (oligomer and fibril) and the gold were represented by the CHARMM 36m force field and solvated with CHARMM-modified TIP3P water molecules. MD simulations were carried out using the Gromacs 2018.4 package with a time step of 2 fs using the Leap frog integrator. Bond lengths to hydrogen in protein were constrained using the LINCS algorithm and water hydrogens were constrained using the SETTLE algorithm. Background ions were added to neutralise full protein formal charges. Long-range electrostatics were treated by the Particle mesh Ewald (PME) method. Protein and non-protein (gold, water, and ions) were coupled separately to an external heat bath (298 K) with the coupling time constant of 1 ps using the velocity rescaling method. All systems were energy minimised, brought to room temperature over 100 ps and equilibrated for 1 ns in constant volume NVT ensemble followed by another 1 ns of constant pressure NPT equilibration with the reference pressure at 1 bar and a time constant of 4 ps using the Parrinello-Rahman barostat. The production runs were carried out for 100 ns for each of the five oligomer–fibril orientations described above in the NPT

ensemble. Structures were saved every 20 ps.

S1.3 MD data analyses

All analyses (main text Fig. 4 and Figs. S1 and S2 below) of interaction energies analyses were performed using Gromacs tools. The maximum protein height profile at gold—water interface was computed by using Tcl/Tk scripting integrated with Visual Molecular Dynamics (VMD).

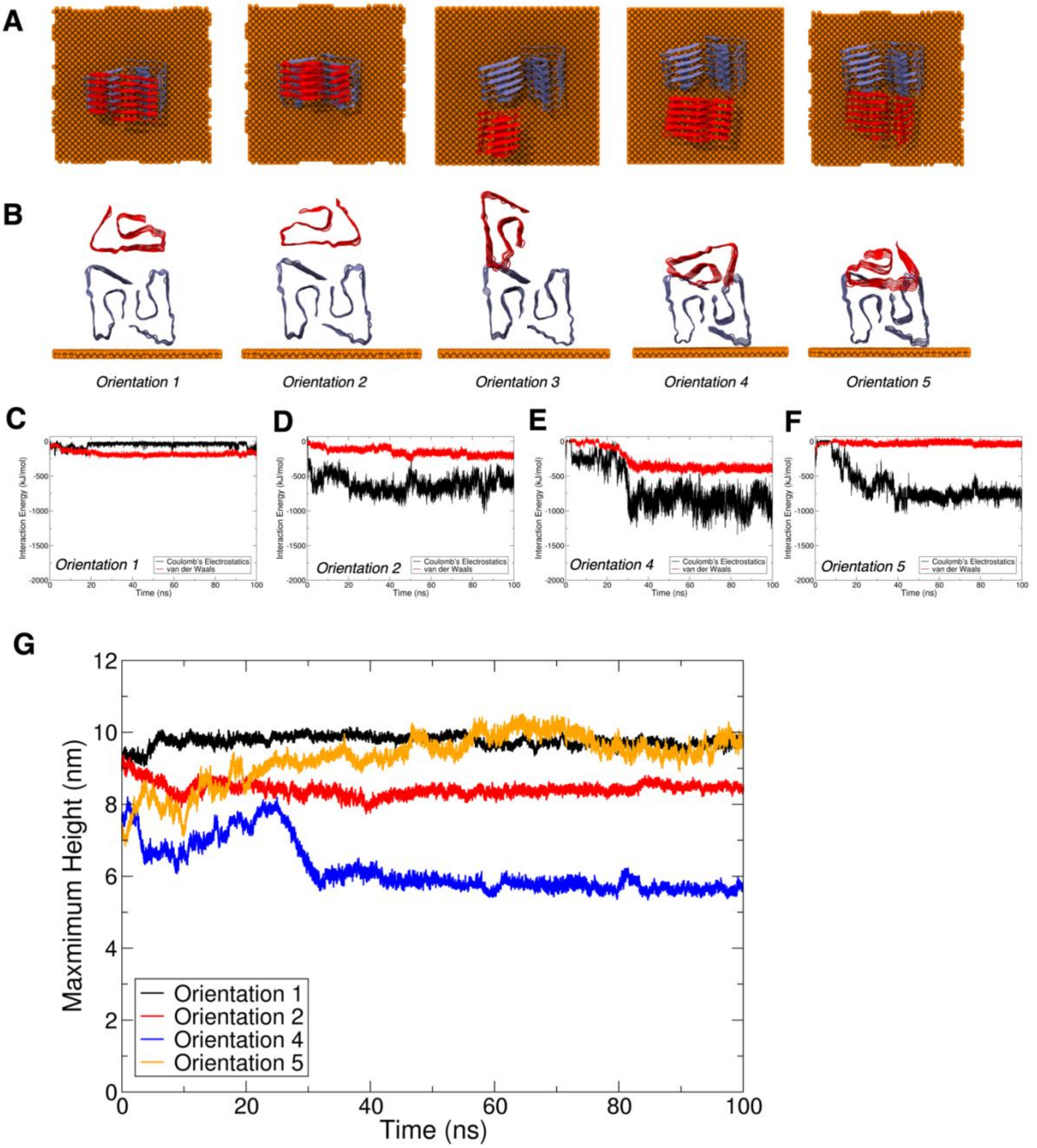

Fig. S1. Initial poses of oligomer–fibril complexes from (A) above and (B) the side. The corresponding final structures after molecular dynamics are given in main text Figure 4. Timelines of oligomer–fibril interaction energies decomposed into electrostatic and van der Waals energies for (C) orientation 1, (D) orientation 2, (E) orientation 4, and (F) orientation 5. (G) Timelines of maximum height profiles for the oligomer-fibril complex for the four binding orientations.

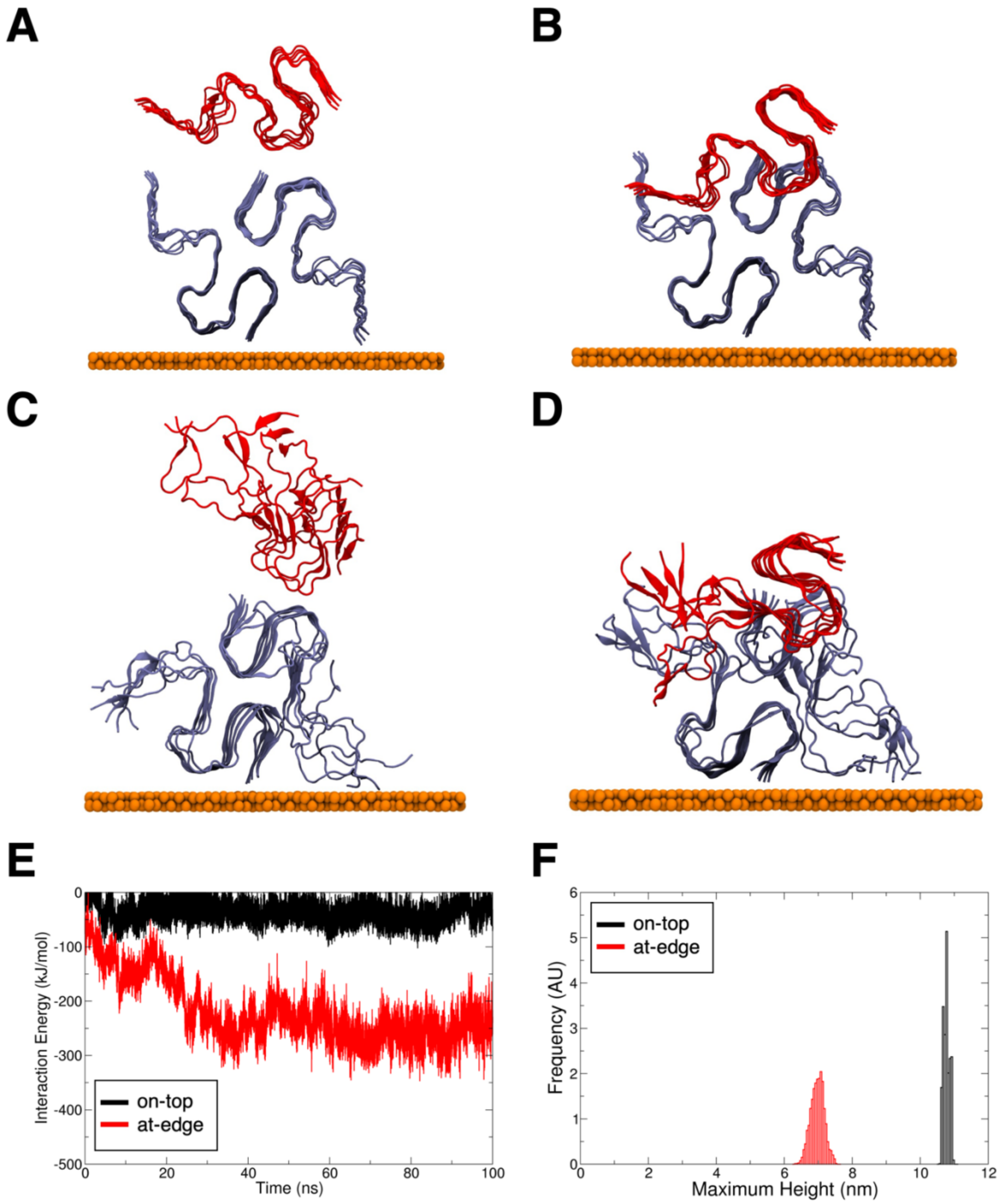

Fig. S2. Control simulations on a control alternative fibril fold, taken from PDB code: 2NAO. The starting oligomer–fibril complexes were built with the oligomer placed either (A) on top or (B) at the edge of the fibril. The corresponding final structures after 100 ns molecular dynamics are shown in (C) and (D). (E) Timelines of oligomer–fibril interaction energies showing the more stable complex formed at the edge of the fibril. (F) Distribution of secondary oligomer height profiles above the gold platform showing predicted mean height of (7.1 ± 0.5) nm in good agreement with the mean secondary oligomer height of (7.9 ± 0.2) nm nm measured by AFM (Fig. 5D) and the height of (8.3 ± 0.6) nm measured in the alternative MD model (Figure 4E).
